# Risk factors for anaemia among pregnant women: A cross-sectional study in Upper East Region, Ghana

**DOI:** 10.1371/journal.pone.0301654

**Published:** 2024-11-14

**Authors:** Clotilda Asobuno, Silas Adjei-Gyamfi, Felix Gumaayiri Aabebe, John Hammond, Chansathit Taikeophithoun, Norbert Ndaah Amuna, Tsunenori Aoki, Hirotsugu Aiga

**Affiliations:** 1 School of Tropical Medicine and Global Health, Nagasaki University, Nagasaki, Japan; 2 Kassena Nankana West District Health Directorate, Ghana Health Service, Paga, Upper East Region, Ghana; 3 School of Clinical Sciences, Auckland University of Technology, Auckland, New Zealand; 4 Savelugu Municipal Hospital, Ghana Health Service, Savelugu, Northern Region, Ghana; 5 Ahafo Regional Health Directorate, Ghana Health Service, Goaso, Ahafo Region, Ghana; 6 Central Regional Health Directorate, Ghana Health Service, Cape Coast, Central Region, Ghana; 7 Faculty of Public Health, University of Health Sciences, Vientiane, Lao PDR; 8 School of Public Health, University of Health and Allied Sciences, Ho, Volta Region, Ghana; MRC Unit The Gambia at LSHTM, GAMBIA

## Abstract

**Background:**

Anaemia in pregnancy (AIP) is a public health concern due to its devastating effects on women and their unborn babies, resulting in increased maternal and neonatal deaths in developing countries. Despite several Ghanaian health policies to combat AIP, AIP is still on the rise. It becomes imperative to identify geographic-specific factors for developing appropriate interventions for the management of AIP. However, Kassena Nankana West District (KNWD) in the Upper East Region of Ghana lacks a study on anaemia risk factors, therefore, this study estimated the prevalence and risk factors for anaemia among pregnant women in the district.

**Methods:**

A cross-sectional study was conducted from February to March 2023 in the KNWD. Approximately 376 pregnant women in their third trimester were randomly selected from 10 health facilities by utilizing the antenatal register as the sampling frame. Anthropometric, obstetric, sociodemographic, and health facility resource characteristics were collected using structured questionnaires and from antenatal records. Mixed-effect logistic regression was used to identify independent factors of anaemia at 95% confidence interval.

**Results:**

Prevalence of AIP was 53.9% (95%CI:48.5%–58.8%). Mild, moderate, and severe anaemia prevalence was 16.9%, 35.3%, and 1.7% respectively. Malaria infection during pregnancy (aOR = 1.64; 95%CI:1.03–2.62) and accessing health facilities without trained laboratory personnel (aOR = 5.49; 95%CI:1.67–18.00) were associated with increased odds of AIP. Belonging to the major ethnic group (aOR = 0.52; 95%CI:0.28–0.85), accessing health facilities without laboratory services (aOR = 0.14; 95%CI:0.04–0.47), and accessing health facilities without sulphadoxine-pyrimethamine drugs (aOR = 0.22; 95%CI:0.06–0.86) in KNWD were also associated with decreased odds of AIP.

**Conclusion:**

KNWD has a severe burden of AIP. Maternal and health facility-related factors were associated with AIP in the district. These factors are preventable. Therefore, the provision of functional laboratory services with dedicated technical personnel, regular supply of sulphadoxine-pyrimethamine drugs to the health facilities, and enhanced community education on malaria prevention are recommended for anaemia control in the district.

## Introduction

Anaemia affects all populations in the world, especially pregnant women, and women in their reproductive age [1]. It is one of the most severe and prevalent nutritional deficiencies of public health concern among pregnant women in low and middle-income countries (LMICs) [2]. The World Health Organization (WHO) describes anaemia in pregnancy (AIP) as decreased haemoglobin (Hb) level of <11g/dL and is further classified into severe, moderate, and mild [3]. Globally, 50,000 young women die each year in pregnancy and childbirth due to anaemia [4]. In sub-Saharan Africa and Southern Asia, anaemia contributes to about 20% of all maternal and perinatal mortalities [3]. Additionally, anaemia does not only reduce work productivity during pregnancy but also leads to adverse pregnancy outcomes such as low birth weight, premature delivery, stillbirths, postpartum haemorrhage, and postpartum morbidities [3, 5–7].

Globally, anaemia is disproportionately distributed and affects about 40.0% to 60.0% of all pregnant women in LMICs [5]. Iron deficiency anaemia which is the commonest type of anaemia, accounts for more than 50.0% of pregnancy-related anaemia cases in LMICs and also affects nearly two billion population across the globe [3, 8]. A multi-level analysis in 10 East African countries disclosed an overall anaemia rate of 41.8%, with a large disparity between specific countries ranging from 23.4% in Rwanda to 57.1% in Tanzania [9]. In Ghana, the Demographic and Health Survey in 2022 estimated anaemia among pregnant women in the country at 51.0% [10] while retrospective studies in the Northern and Upper East Regions of the country reported anaemia prevalence of 45.3% and 50.8% respectively [11, 12]. Data from the Kassena-Nankana West District Health Directorate indicates that, from 2018 to 2021, the proportion of registrants with anaemia during antenatal care significantly increased from 33.0% to 46.0%. Moreover, during the same period, the trend prevalence of anaemia in the third trimester of pregnancy in the district gradually upsurged from 31.0% to 50.9% [13]. This highlights the need to conduct studies to identify factors associated with anaemia in the district.

Despite numerous policy efforts and ongoing interventions that have been proven to have an affirmative impact on the prevention and control of anaemia in most developing countries such as the provision of chemoprophylaxis treatment including anthelmintics, vector elimination via the distribution of insecticide-treated bed nets [1, 3, 8, 11], and supplementation of iron folic acid tablets [3, 4], anaemia is still on the rise [11, 14, 15]. Additionally, Ghana has specifically benefited from the Scaling Up Nutrition (SUN) Movement (Strategy 3.0) which has created a productive impact on nutrition through education, water and hygiene practices, and environmental sanitation, as well as strengthening food, health, and social protection systems [16] in forestalling anaemia among pregnant mothers.

The contributing factors of anaemia in pregnancy are largely classified into individual, cultural, nutritional, socioeconomic, and infection-related factors [5, 8]. Some reported studies in LMICs regarded human immunodeficiency virus infection, primigravida, multiparity, and teenage pregnancies as prominent determinants of anaemia during pregnancy [5, 11, 17–20]. In Africa and Ghana, some predictors of anaemia during gestation are elderly pregnant women, malaria infection, low household wealth index, and illiteracy (no formal education) [5, 15, 21, 22]. In the Kassena-Nankana West District (KNWD), relevant scientific studies on risk factors of pregnancy-related anaemia for informing appropriate interventions are lacking. It is not clear whether there are some service delivery factors, inherent maternal factors, and lack of client adherence to medications that are leading to the persistent rise in anaemia among these pregnant women. Notwithstanding, most studies in Ghana do not consider health facility-related determinants of anaemia for making informed decisions [11, 14, 22]. Hence, there is a pressing need to investigate health facility-based and maternal determinants or factors that could be associated with anaemia during the period of pregnancy. This study therefore seeks to estimate the prevalence and risk factors of anaemia among pregnant women in the KNWD, Upper East Region (UER) of Ghana.

## Materials and methods

### Study design

This study was a facility-based cross-sectional type conducted to estimate anaemia prevalence and identify the risk factors for anaemia among pregnant women in KNWD of Upper Eastern Ghana.

### Study area

The study was conducted at KNWD in the UER of Ghana. KNWD was carved out of Kassena-Nankana Municipality in 2007 and was launched on 29^th^ February 2008. This new district’s 2021 population projected from the National Population and Housing Census was 89,043 with a yearly growth rate of 2.4% [23]. KNWD shares a border with Bulsa District in the southwest, Sisala District in the west, and Burkina Faso in the north, Bongo and Bolgatanga Districts in the east and northeast respectively. Kassenas and Nankanas are the predominant ethnic groups in the district, with the citizens mostly being farmers and small-scale traders who rely on dry and rainy climatic seasons annually. There are nine health centers, one district hospital as a referral center, 51 CHPS compounds/zones, and three private health facilities in KNWD [13]. These facilities provide health services to all 115 communities in the district. The total number of women in their reproductive age is estimated at 21,370 (24%), with yearly estimated pregnancies of 3,562 (4%) [23]. Furthermore, the total number of antenatal care (ANC) visits in 2021 was 17,566, of which the ANC services were daily provided by 10 health facilities. In the same year, the rate of sulphadoxine-pyrimethamine (SP) intake of at least three doses was approximately 90.0% [13]. In Ghana, the free maternal healthcare policy mandates all health facilities to monitor or test the Hb of all pregnant women at registration, 28 weeks, and 36 weeks of gestation as part of the routine antenatal services [12, 24–26]. Hence, in 2021, the coverage of Hb checks in the third trimester of pregnancy in the district was nearly universal [13]. Nonetheless, due to the frequent increase in ANC attendance, facility-trained staff complement the work of the 11 professional laboratory scientists by testing the Hb of pregnant women across the various health facilities in the district.

### Study population

This study interviewed 395 pregnant women who were registered and attending antenatal care in the district. Pregnant women who were in their third trimester and had maternal and child health record books (MCHRBs) were included in the study. Those with haematological problems or who were seriously sick during data collection were excluded from the study.

### Sample size and sampling methods

The sample size (n) was calculated using the Cochran formula [27]; $\text{n}=\frac{{Z_{\propto /2}}^{2}\;\text{x}\;\text{p}(1-\text{p})}{{\text{d}}^{2}}$.

The study employed the prevalence (p) of AIP in a neighbouring district (Kassena Nankana East Municipal) in the UER of Ghana estimated at 42.7% [14], margin error (d) of 5%, and standard normal variate ($Z_{\propto /2}$) at 95% confidence level of 1.96, the sample size was initially estimated at 376. Due to incomplete responses, 5% of the sample size was added [7] to produce an approximated final sample size of 395 (= 376/ [1–0.05]).

Of the 64 health facilities in the KNWD, only 10 health facilities provide daily ANC services in the district. During sampling, these 10 major health facilities were selected for the study. Probability proportional to size technique was used to determine the sample sizes for each health facility. Each facility’s ANC register was obtained to line-list all pregnant women who were eligible to form a sampling frame. Thereafter, a simple random sampling method was used to select the respondents from each health facility. The addresses (including contact numbers) of the recruited respondents were then taken to trace these respondents to their homes.

### Data collection

A pretested structured questionnaire was used to collect the data from the selected health facilities and pregnant women in the comfort of their homes from 13^th^ February to 15^th^ March 2023. The questionnaire was designed using Kobo collect. Data was first collected on health facility’s resources for anaemia measurement and prevention including laboratory services on blood testing, and availability of iron and folic acid (IFA) supplements, SP drugs, and MCHRBs. The next phase of the data collection was at the households of respondents. Before household data collection commenced, the trained research assistants (public health officers) obtained the addresses and contacts (telephone numbers) of respondents and/or representatives from the ANC register. The midwives in each facility helped the research assistants to obtain this information. The research assistants (who received one week of intensive training) then visited the homes of the recruited respondents after booking an appointment with them. Data on Hb levels, antenatal visits, anthropometry, anthelminthic drugs intake, SP doses intake, human immunodeficiency virus (HIV) infection, and malaria infection during pregnancy were extracted from the MCHRBs, and other information including sociodemographic, obstetric, IFA intake, and socioeconomic characteristics were collected through observation and structured interviews.

### Study variables

The dependent variable for this study was AIP in the third trimester classified as; (i) Anaemia (Hb: < 11 g/dl) and (ii) No anaemia (Hb: ≥ 11 g/dl). Anaemia status was further classified into mild (Hb: 10.0–10.9g/dl), moderate (Hb: 7–9.9 g/dl), and severe anaemia (Hb: < 7 g/dl) [3].

The sociodemographic, socioeconomic, anthropometric, antenatal, obstetric, and availability of resources for anaemia measurement and prevention at the health facilities were the independent variables. Most of the independent variables including iron-folic acid intake, anthelminthic drugs intake, SP doses intake, human immunodeficiency virus (HIV) infection, malaria during pregnancy, and availability of health facility resources (SP drugs, iron-folic acid tablets, anthelmintics, antimalaria drugs, functional laboratory machines, and trained laboratory personnel) were categorized as “Yes” or “No”. Pre-pregnancy (first-trimester) BMI was classified into underweight (< 18.5 kg/m^2^), normal (≥ 18.5 to 24.9 kg/m^2^), and overweight/obese (≥ 25.0 kg/m^2^) using the WHO classification [28]. Gravida was grouped into primigravida (0–1 pregnancy) and multigravida (≥ 2 pregnancies). Parity was classified into primipara (0–1 delivery) and multipara (≥ 2 deliveries) [6]. Based on maternal gestational age, ANC initiation was summarized into first [≤ 13 weeks), second (14–27 weeks), and third (28–41 weeks) trimesters [28]. By employing principal component analysis, the wealth index of respondents was assessed using household properties and housing quality which was used as a proxy indication for socioeconomic status of the respondents. The wealth index was then categorized into five quintiles namely: poorest, poorer, middle, richer, and richest [6]. Other variables such as ethnic group, religion, educational level, occupation, marital status, and household size were also summarized and categorized based on previous studies [1, 6, 7, 12].

### Statistical analysis

Statistical analysis was conducted using STATA version 17.0 (Stata Corporation, Texas, USA) with all analyses determined at 95% confidence level. Chi-square or Fisher’s exact test was used to determine the bivariate association between anaemia status and each independent variable (individual-level and health facility-level characteristics). Multicollinearity was checked among these predictor variables before employing them in the multivariate analyses. Variance Inflation factor (VIF) was used during multicollinearity testing. Since all the variables had VIF less than 10 during the testing, they were all included in the multivariate analyses [6, 12]. In the multivariate analysis, a two-level mixed-effect logistic regression model was used to identify factors associated with AIP [29, 30]. Individual-level and facility-level variables that showed a significant bivariate relationship with anaemia were used for the multivariate analysis. Four models were estimated using the maximum likelihood approach while the final model (model IV) was compared to that of the null model (model I). Two standard metrics (intraclass correlation coefficient [ICC], and proportional change in variance [PCV]) were used to decompose and highlight the magnitude of variability in the predictors of anaemia across the health facilities. The models were compared using measures of goodness of fit via the Akaike information criterion (AIC) values. The model with the smallest AIC value was regarded as the best-fitted model [9, 31–33].

### Ethical considerations

The study obtained ethical clearance from the Nagasaki University School of Tropical Medicine and Global Health (TMGH) Institutional Review Board (approval number:NU_TMGH_2022_225_1), and the Ghana Health Service Ethics Review Committee (approval number: GHS-ERC:023/12/22). Permission was obtained from the UER Health Directorate, KNWD Health Directorate, and Heads of the health facilities before the data was collected. Written informed consent was obtained from all pregnant women above 18 years of age while informed assent was used to attain clearance from the parents/guardians of those below 18 years of age after they were given information on the purpose of the study and what was required of them as study participants.

## Results

### Socio-demographic, antenatal, and obstetric characteristics of study participants

A total of 395 respondents were interviewed. However, 19 incomplete questionnaires were found during data cleaning. Hence, 376 respondents were used for the data analysis. The ages of the pregnant women ranged from 16 to 50 years, with a mean age of 27 years (sd = 6.3). A greater number of the respondents were married (93.0%) and were Christians (85.0%). The respondents were predominantly from the Kassena ethnic group (55.0%), while above half of them were unemployed (52.0%). About 10.0% of them had no formal education (Table 1). More than one-quarter of the women (27.0%) received IFA tablets from their health facilities. While 76.0% of the pregnant women slept under ITNs the night before the interview, one-quarter of them (25.0%) had taken at least four doses of SP during pregnancy. Slightly above one-third of the women (35.0%) experienced malaria during their pregnancy period (Table 2).

**Table 1:** Sociodemographic characteristics of respondents and prevalence of anaemia (n = 376).

**Variables**		**Frequency distribution**		**Bivariate analysis**		
		**Frequency (n)**	**Proportion (%)**	**Anaemia n (%)**	**No anaemia n (%)**	**p-value ^¶^**
**Age group (years) (mean = 27.13; sd = 6.28)**						
	< 20 years	47	12.5	20 (11.5)	28 (13.9)	0.779
	20–35 years	298	79.3	139 (79.8)	158 (78.1)	
	≥ 35 years	31	8.2	15 (8.7)	16 (8.0)	
**Marital status**						
	Married	350	93.1	166 (95.4)	183 (90.6)	0.135
	Single	24	6.4	8 (4.6)	17 (8.4)	
	Widowed	2	1.2	0 (0.0)	2 (1.0)	
**Educational level**						
	No education	37	9.8	19 (10.9)	18 (8.9)	0.276
	Primary school	85	22.6	37 (21.2)	49 (24.3)	
	JHS/middle school	169	45.0	73 (42.0)	96 (47.5)	
	SHS/vocational school	62	16.5	36 (20.7)	26 (12.9)	
	Tertiary	22	5.9	9 (5.2)	13 (6.4)	
**Ethnicity**						
	Kassena	207	55.1	54 (31.0)	101 (50.0)	0.002*
	Nankana	155	41.1	111 (63.8)	96 (47.5)	
	Bulsa	4	1.1	3 (1.7)	1 (0.5)	
	Others (French, Akan)	10	2.7	6 (3.5)	4 (2.0)	
**Religion**						
	Christianity	320	85.1	28 (16.1)	18 (8.9)	0.070
	Islam	46	12.2	143 (82.2)	177 (87.6)	
	Traditionalist	10	2.7	3 (1.7)	7 (3.5)	
**Occupation**						
	Unemployed	194	51.6	95 (54.6)	100 (49.5)	0.589
	Self-employed	165	43.6	71 (40.8)	93 (46.0)	
	Public/civil servant	17	4.5	8 (4.6)	9 (4.5)	
**Household size (mean = 6.39; sd = 2.69)**						
	< 10 persons	322	85.6	149 (85.6)	173 (85.6)	0.997
	≥ 10 persons	54	14.4	26 (14.4)	29 (14.4)	
**Wealth index**						
	Poorest	75	19.9	35 (20.2)	40 (19.8)	0.789
	Poorer	75	19.9	34 (19.5)	41 (20.3)	
	Middle	75	19.9	38 (21.8)	37 (18.3)	
	Rich	75	19.9	26 (14.9)	39 (19.3)	
	Richest	76	20.4	41 (23.6)	45 (22.3)	
**Haemoglobin levels (g/dl) (mean = 10.9; sd = 1.4)**		**Frequency (n)**	**Proportion (%)**	**95%CI**		
	No anaemia	172	46.1	41.2–51.5%		
	Anaemia	204	53.9	48.5–58.8%		
	Mild anaemia	64	16.9	13.1–20.9%		
	Moderate anaemia	133	35.3	30.5–40.4%		
	Severe anaemia	7	1.7	1.1–3.4%		

*p-value < 0.05

^**¶**^ Chi-square/Fisher’s exact test

**Table 2:** Antenatal and obstetric characteristics of respondents (n = 376).

Variables		Frequency distribution		Bivariate analysis		
		Frequency (n)	Proportion (%)	Anaemia n (%)	No anaemia n (%)	p-value ^¶^
**Pre-pregnancy BMI (mean = 22.77 kg/m²; sd = 4.61)**						
	Underweight (BMI < 18.5)	22	5.9	5 (2.9)	10 (5.0)	0.182
	Normal BMI (BMI ≥ 18.5 to 24.9)	277	73.7	127 (73.0)	157 (77.7)	
	Overweight/obesity (BMI ≥ 25.0)	77	20.5	42 (24.1)	35 (17.3)	
**Pre-pregnancy weight (mean = 59.70 kg; sd = 12.42)**						
	< 50 kg	6	1.60	12 (6.9)	16 (7.9)	0.706
	≥ 50 kg	370	98.40	162 (93.1)	186 (92.1)	
**Pre-pregnancy height (mean = 159.37 cm; sd = 21.39)**						
	< 150 cm	16	4.3	4 (2.3)	6 (3.0)	0.687
	> 150 cm	360	95.7	170 (97.7)	196 (97.0)	
**Gestational age for first ANC visit**						
	1st trimester	152	40.4	71 (40.8)	83 (41.1)	0.334
	2^nd^ trimester	211	56.1	102 (58.6)	114 (56.4)	
	3^rd^ trimester	13	3.5	1 (0.6)	4 (2.5)	
**IFA intake during pregnancy**						
	Intake	363	96.5	165 (94.8)	198 (98.0)	0.091
	No intake	13	3.5	9 (5.2)	4 (2.0)	
**IFA intake 24 hours before interview**						
	intake	336	89.4	153 (87.9)	183 (90.6)	0.404
	No intake	40	10.6	21 (12.1)	19 (9.4)	
**Source of IFA supplements**						
	Health facility	101	26.8	53 (31.0)	48 (23.8)	0.114
	Chemical shop	275	73.2	121 (69.0)	154 (76.2)	
**ITNs use during pregnancy**						
	No ITN use	33	8.8	161 (92.5)	182 (90.1)	0.406
	ITN use	343	91.2	13 (7.5)	20 (9.9)	
**ITNs use night before interview**						
	ITN use	285	75.8	138 (79.3)	147 (72.8)	0.140
	No ITN use	91	24.2	36 (20.7)	55 (27.2)	
**Number of SP intake**						
	None	34	9.0	12 (6.9)	21 (10.4)	
	1–3 doses	284	75.5	121 (69.5)	130 (64.4)	0.409
	> 3 doses	92	24.5	41 (23.6)	51 (25.3)	
**Anthelminthic intake**						
	Intake	234	62.2	63 (36.2)	79 (39.1)	0.563
	No intake	142	38.0	111 (63.8)	123 (60.9)	
**Nutrition counselling**						
	Received	337	89.6	157 (90.2)	180 (89.1)	0.722
	Not received	39	10.4	17 (9.8)	22 (10.9)	
**Gravida**						
	Primigravida (0–1 pregnancies)	79	21.0	41 (23.6)	38 (18.8)	0.259
	Multigravida (> 2 pregnancies)	297	78.9	133 (76.4)	164 (81.2)	
**Parity**						
	Primipara (0–1 deliveries)	177	47.1	89 (51.1)	88 (43.6)	0.142
	Multipara (> 2 deliveries)	199	52.9	85 (48.9)	144 (56.4)	
**Malaria episode**						
	Episode	131	34.8	48 (27.6)	83 (41.1)	0.006*
	No episode	245	65.2	126 (72.4)	119 (58.9)	
**HIV infection (n = 372)**						
	With HIV	4	1.1	0 (0.0)	4 (1.9)	0.062
	Without HIV	368	97.9	174 (100.0)	198 (97.1)	
**Worm Infestation (n = 173)**						
	With infestation	3	1.7	63 (36.2)	79 (39.1)	0.075
	Without infestation	170	98.3	111 (63.8)	123 (60.9)	

* p-value < 0.05

^**¶**^ Chi-square/Fisher’s exact test

### Prevalence of anaemia among study participants

The mean haemoglobin level was found to be 10.9 g/dl (sd = 1.4). The prevalence of anaemia was 53.9% (95%CI:48.5%–58.8%) which was classified into mild, moderate, and severe anaemia corresponding to 16.9%, 35.3%, and 1.7% respectively (Table 1).

### Availability of health facilities’ resources for anaemia measurement and prevention during antenatal visits

Over half of the pregnant women made ANC visits to health facilities without functional laboratory services (54.0%), while those who attended health facilities without haemoglobin testing machines were nearly one-third (30.0%). A greater proportion of pregnant women received ANC services from health facilities without trained laboratory personnel (59.0%). More than half of the pregnant women (62.0%) accessed the health facilities having no stock of MCHRBs. Furthermore, 79.0% of the respondents accessed ANC in the health facilities having no stock of IFA supplements. Few pregnant women (12.0%) did not receive SP drugs from facilities they visited for ANC services (Table 3).

**Table 3:** Availability of health facilities’ resources for anaemia measurement and prevention during antenatal visits (n = 376).

Variables		Frequency distribution		Bivariate analysis		
		Frequency (n)	Proportion (%)	Anaemia n (%)	No anaemia n (%)	p-value ^¶^
**Accessing health facilities without laboratory services**						
	Without laboratory services	201	53.5	70 (40.2)	105 (52.0)	0.023*
	With laboratory services	175	46.5	104 (59.8)	97 (48.0)	
**Accessing health facilities without haemoglobin testing machines**						
	Without testing machines	114	30.3	111 (63.8)	151 (74.7)	0.037*
	With testing machines	262	69.7	63 (36.2)	51 (25.3)	
**Accessing health facilities without trained laboratory personnel**						
	Without trained personnel	223	59.3	73 (41.9)	80 (39.6)	0.024*
	With trained personnel	153	40.7	101 (58.1)	122 (60.4)	
**Accessing health facilities without MCHRB**						
	Without MCHRBs	234	62.2	57 (32.8)	83 (41.1)	0.096
	With MCHRBs	142	37.8	117 (67.2)	119 (58.9)	
**Accessing health facilities without IFA supplements**						
	Without IFA	296	78.7	40 (23.0)	40 (19.8)	0.452
	With IFA	80	21.3	134 (77.0)	162 (80.2)	
**Accessing health facilities without antimalaria drugs**						
	Without malaria drugs	0	0	0 (0.0)	0 (0.0)	1.000
	With malaria drugs	376	100.0	202 (100)	174 (100)	
**Accessing health facilities without anthelminthics**						
	Without dewormer	376	100.0	0 (0.0)	0 (0.0)	1.000
	With dewormer	0	0	202 (100)	174 (100)	
**Accessing health facilities without SP drugs**						
	Without SP	44	11.7	158 (90.8)	174 (86.1)	0.040*
	With SP	332	88.3	16 (9.2)	28 13.9)	

*p-value < 0.05

^**¶**^ Chi-square/Fisher’s exact test

### Association of anaemia with exposure variables

As shown in Tables 1–3, individual-level variables including ethnicity (p = 0.002), malaria during pregnancy (p = 0.006), and facility-level variables like accessing health facilities without laboratory services (p = 0.023), accessing health facilities without haemoglobin testing machines (p = 0.021), accessing health facilities without trained laboratory personnel (p = 0.024), and accessing health facilities without SP drugs (p = 0.040) were associated with anaemia at the bivariate level.

### Random effect and model comparison

As presented in Table 4, the random effect or facility-level variation of anaemia and model comparison are elucidated. The variance component analysis was conducted to disentangle the total variance of anaemia as evidenced in the null model (model I). The facility was used as a level two variable. It implies that, the facility-level variance which indicates the total variance of anaemia that can be attributed to the context of the facility in which the women were attending antenatal care services was estimated. This analysis was accounted for by the significance of the facility-level variance [facility variance = 1.22; standard error (SE) = 0.15; p = 0.002], exhibiting the existence of statistically significant differences between facilities regarding AIP. This was further reinforced by the ICC in the null model which showed that about 64.91% of the variation of AIP was attributed to the difference at facility-level factors. Likewise, the final model (model IV) indicates that about 39.29% of the variation of AIP was attributable to both the individual-level and facility-level factors. Considering the model comparison, AIC was used to determine model fitness. Subsequently, the model with the lowest AIC value (Model IV) was considered to be the best-fitted model.

**Table 4:** Associated factors for anaemia in pregnancy.

Variables	Model I (Null)	Model II	Model III	Model IV
	a0R (95%CI)	a0R (95%CI)	a0R (95%CI)	a0R (95%CI)
***Individual-level variables***				
**Ethnicity**				
Kassena	–	0.58 (0.32–0.99)	–	**0.52 (0.28–0.95)** *
Bulsa	–	0.20 (0.02–2.10)	–	0.15 (0.01–1.66)
Others (French, Akan)	–	0.49 (0.12–1.99)	–	0.49 (0.12–2.11)
Nankana	–	Reference	–	Reference
**Malaria episode in pregnancy**				
Episode	–	1.83 (1.16–2.87)	–	**1.64 (1.03–2.62)** *
No episode	–	Reference	–	Reference
***Facility-level variables***				
**Accessing health facilities without laboratory services**				
Without laboratory services	–	–	0.15 (0.04–0.48)	**0.14 (0.04–0.47)** *
With laboratory services	–	–	Reference	Reference
**Accessing facilities without haemoglobin testing machines**				
Without testing machine	–	–	0.82 (0.49–1.34)	0.85 (0.51–1.41)
With testing machine	–	–	Reference	Reference
**Accessing facilities without trained laboratory personnel**				
Without trained personnel	–	–	5.54 (1.70–18.02)	**5.49 (1.67–18.00)** *
With trained personnel	–	–	Reference	Reference
**Accessing health facilities without SP drugs**				
Without SP drugs	–	–	0.22 (0.06–0.87)	**0.22 (0.06–0.86)** *
With SP drugs	–	–	Reference	Reference
**Random parameters**	**Model I**	**Model II**	**Model III**	**Model IV**
Facility level variance (SE)	1.22 (0.15)+	1.13 (0.12)+	1.25 (0.17)+	1.13 (0.12)+
ICC (%)	64.91	58.23	73.06	57.87
PCV (%)	Reference	23.57	23.60	39.29
**Model fit statistics**				
AIC	510.88	506.76	500.46	497.51

* p-value < 0.05

+ p-value < 0.01 aOR: Adjusted odds ratio, Bold = Significant aOR and 95CI%, AIC: Akaike information criterion, ICC: Intraclass correlation coefficient, PCV: Proportional change in variance, SE: Standard deviation

### Associated risk factors for anaemia in pregnancy

Table 4 also exhibits the associated factors of anaemia in pregnancy. The findings suggest that women who had malaria infection during pregnancy had 1.64 times increased odds of getting anaemia (aOR = 1.64; 95%CI:1.03–2.62). Pregnant women belonging to the major ethnic group (Kassena) had 48.0% reduced odds of anaemia (aOR = 0.52; 95%CI:0.28–0.85). Additionally, pregnant women who accessed health facilities without laboratory services (aOR = 0.14; 95%CI:0.04–0.47) and without stock of SP drugs to supply to pregnant women (aOR = 0.22; 95%CI:0.06–0.86) had decreased odds of anaemia. Lastly, pregnant women who visited health facilities without trained laboratory personnel were more likely to be anaemic (aOR = 5.49; 95%CI:1.67–18.00).

## Discussion

AIP is a global public health concern due to its consequences on the lives of women and unborn babies such as low birth weight, preterm births, stillbirths, and maternal deaths [6, 7, 34, 35]. The World Health Assembly aims to reduce anaemia by 50% in 2025 [3], which motivates most countries to identify and address factors responsible for anaemia during pregnancy. Hence, this facility-based cross-sectional study was conducted to estimate anaemia prevalence and the possible risk factors among pregnant women in KNWD.

This study’s mean haemoglobin level was 10.9g/dl with anaemia prevalence of 53.9%. Our study’s anaemia prevalence was higher than the global and East African prevalence of 41.8% [9, 36] and the prevalence reported by the 2022 Ghana Demographic and Health Survey (51%), Sunyani municipality (40.8%), and Hohoe municipality (32.7%) of Southern Ghana [10, 37, 38]. Furthermore, previous studies in Northern Ghana reported a similar prevalence to that of the present study [5, 11] while a similar study in Northern Tanzania reported higher anaemia rate of 81.3% [39]. Paired with this study’s results, diversities in the cultural settings, beliefs, and economic activities between the southern and northern parts of Ghana and across Africa might be the reason for variations in the anaemia prevalence. Likewise, the variations in anaemia status among participants could be explained by several factors including the type of ethnic group, malaria infection, and accessing health facilities without laboratory services, trained laboratory personnel, and SP drugs which were found to be statistically associated with anaemia during pregnancy in this study.

Women belonging to the major ethnic group (called Kassena) in the KNWD had decreased odds of anaemia during pregnancy. This study result is at variance with that of cross-sectional surveys in Wa and Savelugu municipalities/districts in Ghana [15, 40] but similar to other study findings in the Negev region of Israel [41] where pregnant mothers belonging to the major ethnic group (known as Bedouin) are less likely to be anaemic. Pregnant women from larger ethnic groups in the Wa and Savelugu municipalities are residentially dispersed and do not necessarily reside in the municipal capitals. Yet, the Kassena ethnic group is mostly found in the capital town of the district, where most women practice petty trading and are economically better off compared to other (minor) ethnic groups in the KNWD [11]. This might influence their food purchasing, food intake, and health-seeking behavior which could reduce the risk of anaemia among the Kassena pregnant women.

The study showed that women with malaria during pregnancy had increased odds of anaemia. Similar to a study in Southern Ghana [38], malaria in pregnancy is associated with anaemia while some cross-sectional studies in Northern Ghana [5, 14] are incongruent. Africa remains the breeding ground for malaria parasites, especially in tropical areas like Ghana [38, 42]. In 2010, malaria contributed to the majority of all deaths and about 11% of maternal deaths in Ghana [42]. Malaria during pregnancy affects the maternal and foetal tissues and breaks down the formation of maternal erythrocytes leading to anaemia [42, 43] and consequently resulting in adverse pregnancy outcomes such as low birth weight and premature deliveries [6, 7, 44]. Ghana adopted WHO-recommended strategies for malaria control such as the distribution of insecticide-treated bed nets and chemoprophylaxis treatment via the intake of SP drugs for the prevention of malaria through the free maternal care policy [22, 45]. However, commitment and adherence to these interventions in KNWD may be suboptimal which could predispose the women to malaria and risk them for anaemia during pregnancy.

Pregnant women who attended ANC services at health facilities without formally trained (professional) laboratory personnel had higher odds of developing anaemia. The present study’s finding is parallel to a similar cross-sectional study in Tanzania [46]. Most of the health facilities in the UER of Ghana do not have adequate formally trained laboratory technicians which propel these facilities to engage or recruit some casual staff (who have no formal training) due to work overload. These casual staff are trained on the job for some time (about 2 to 3 months) and are made to assume full responsibility at the laboratory units especially when the formally trained laboratory technician is transferred to another higher facility or observes his/her official leave of absence (study, annual or maternity leave). Hence, this finding could occur as these untrained and/or informally trained laboratory persons are more likely to wrongly read, interpret, and report laboratory results due to a lack of technical expertise which may risk pregnant women from receiving the appropriate interventions for possible anaemia detection.

Our study revealed that pregnant women who accessed ANC services at health facilities that do not provide laboratory services were less likely to develop anaemia. This unexpected finding could happen as health professionals in the facilities without laboratory services do not pay much attention to the Hb testing of these pregnant women and may not know the Hb status (being anaemic or non-anaemic) of the women. However, these women may be given nutrition education and counseling in addition to strict monitoring of IFA supplementation and compliance which could indirectly render some form of protection against anaemia. On the other hand, laboratory services are essential for the early detection, diagnosis, and monitoring of anaemia in pregnancy. These services facilitate targeted interventions, such as micronutrient supplementation and nutritional counseling, which are vital in preventing maternal and foetal complications associated with anaemia [22, 47, 48]. Timely and appropriate use of laboratory tests significantly contributes to the overall better health outcomes of pregnant women and their unborn foetus. Despite the enormous importance of the availability of laboratory services in curbing anaemia in pregnancy, majority of the health facilities in KNWD do not have the basic laboratory equipment to conduct the services. As a result, most women delay their subsequent antenatal visits due to referral for laboratory services including Hb tests, and may unwittingly resort to appropriate nutrition practices which could indirectly reduce the risk of anaemia. Although the health facilities without laboratory services in KNWD reduced the odds of anaemia during pregnancy, it does not change the fact that the district health management must ensure the urgent provision of laboratory services in all the health facilities to enhance universal health coverage.

Lastly, women attending health facilities without SP drugs unexpectedly had lower odds of anaemia during pregnancy which is inconsistent with a report from McClure and his colleagues who asserted that health facilities with a higher stock of SP drugs reduced the risk of maternal anaemia [49]. SP drugs are religiously known for their efficacy in preventing and treating malaria during pregnancy, which serves as a significant risk factor for anaemia [42, 45]. However, pregnant women accessing health facilities with no stock of SP drugs in KNWD could benefit from other preventive measures like the use of ITNs and indoor residual spraying in the KNWD which could protect these pregnant women from malaria and subsequently anaemia [22, 45]. As much as facilities without SP drugs may be an indirect contributor to anaemia in pregnancy, efforts must be put in place to prevent regular stock-out of SP drugs across the health facilities in the KNWD. Additionally, further studies should consider health facilities’ impact on anaemia by assessing the SP stock levels.

### Limitations of the study

Sampling bias could occur due to the exclusion of pregnant women who lost or do not have MCHRBs because the study collected some of the data directly from the MCHRBs. Additionally, pregnant women who were not in their third trimester were excluded from this study which could affect sampling and anaemia prevalence. This study collected secondary data on haemoglobin levels, obstetric, and anthropometric characteristics documented in the MCHRBs as some of the data may be inaccurate due to mis-recording or mismeasurement leading to information bias. Information on other significant predictors of anaemia such as dietary diversity, frequency of and compliance to IFA intake, and sanitation variables were not collated nor investigated which might affect the findings of our study. The study could not be generalized and may need a larger sample size to determine rare risk factors of anaemia. Lastly, since it is a cross-sectional study, it might not measure the seasonal findings of AIP.

## Conclusion

KNWD has a severe burden of AIP which is affected by maternal major ethnic group, malaria during pregnancy, and health facilities-related factors. Enhancing malaria prevention and treatment measures is essential for addressing some of these factors in KNWD. Ghana Health Service should recruit more laboratory personnel and periodically conducts in-service training to update the staff on haemoglobin testing. Moreover, Ghana Health Service should provide health facilities in hard-to-reach communities and peripherals with well-equipped laboratories for testing haemoglobin and other laboratory tests to increase accessibility for minor ethnic groups and provide a conducive environment for anaemia treatment protocol. We recommend that future larger studies should be conducted to assess health facilities-related factors on anaemia during pregnancy.

## Supporting information

S1 Dataset Datasets collected and analyzed for the study. (DTA)

## Abbreviations

ANC: Antenatal care
AIP: Anaemia in pregnancy
BMI: Body mass index
CHPS: Community-based preventive services
Hb: Haemoglobin
HIV: Human immunodeficiency virus
IFA: Iron and folic acid
ITNs: Insecticide-treated bed nets
KNWD: Kassena Nankana West District
LMICs: Low-and middle-income countries
MCHRBs: Maternal and child health record books
SP: Sulphadoxine pyrimethamine
SUN: Scaling Up Nutrition
UER: Upper East Region
